# Pro-Tumorigenic and Thrombotic Activities of Platelets in Lung Cancer

**DOI:** 10.3390/ijms241511927

**Published:** 2023-07-25

**Authors:** Ronald Anderson, Bernardo L. Rapoport, Helen C. Steel, Annette J. Theron

**Affiliations:** 1 Department of Immunology, Faculty of Health Sciences, University of Pretoria, Pretoria 0001, South Africa; bernardo.rapoport@up.ac.za (B.L.R.); helen.steel@up.ac.za (H.C.S.); atheron@up.ac.za (A.J.T.); 2 The Medical Oncology Centre of Rosebank, Johannesburg 2196, South Africa

**Keywords:** immunosuppression, lung cancer, lung-cancer-associated thrombosis, platelets, platelet-derived vesicles, platelet factor 4, transforming growth factor beta 1, tissue factor

## Abstract

Aside from their key protective roles in hemostasis and innate immunity, platelets are now recognized as having multifaceted, adverse roles in the pathogenesis, progression and outcome of many types of human malignancy. The most consistent and compelling evidence in this context has been derived from the notable association of elevated circulating platelet counts with the onset and prognosis of various human malignancies, particularly lung cancer, which represents the primary focus of the current review. Key topics include an overview of the association of lung cancer with the circulating platelet count, as well as the mechanisms of platelet-mediated, pro-tumorigenic immunosuppression, particularly the role of transforming growth factor beta 1. These issues are followed by a discussion regarding the pro-tumorigenic role of platelet-derived microparticles (PMPs), the most abundant type of microparticles (MPs) in human blood. In this context, the presence of increased levels of PMPs in the blood of lung cancer patients has been associated with tumor growth, invasion, angiogenesis and metastasis, which correlate with disease progression and decreased survival times. The final section of the review addresses, firstly, the role of cancer-related platelet activation and thrombosis in the pathogenesis of secondary cardiovascular disorders and the associated mortality, particularly in lung cancer, which is second only to disease progression; secondly, the review addresses the potential role of antiplatelet agents in the adjunctive therapy of cancer.

## 1. Introduction

There is an increasing awareness of the threat posed by platelets to patients with certain types of cancer, particularly colon, lung, ovarian and stomach cancers [1,2]. Platelets are not only implicated in the various stages of tumorigenesis but also play a role in the pathogenesis of the often-fatal cardiovascular events (CVEs) associated with malignant diseases, particularly lung cancer. The current review is primarily focused on the role of platelets in the pathogenesis of lung cancer and the development of the associated CVEs, especially the probable critical involvement of transforming growth factor-β1 (TGF-β1). In addition, the converse situation in which pre-existing CVEs may predispose the human body to the emergence of lung cancer, as well as representing a potential impediment to anti-cancer therapy, is briefly reviewed. The final section of the review covers the various types of potential adjunctive anti-platelet therapies, as well as the potential risks and benefits associated with some of these strategies.

## 2. Association of Increased Platelet Counts with the Development of and Mortality from Solid Malignancies

The putative involvement of platelets in the pathogenesis of various types of solid malignancy has been recognized for several decades, largely based on the measurement of complete full blood counts, which revealed associations with high numbers of circulating platelets. In this context, two recent studies reported that the transition from a moderate to an elevated/very high platelet count preceded the diagnosis of a solid malignancy [1,2]. The first of these was a prospective cohort study, in which the one-year cancer incidence was estimated in a cohort of 295,312 primary-case English participants aged >40 years and with no prior cancer diagnoses. Data spanning the period from May 2005 to April 2014 for England was sourced from the UK Clinical Practice Research Datalink, the GOLD database and the National Cancer Registration and Analysis Service [1]. Increased rates of cancer associated with above-average platelet counts were most prominent in male participants aged >60 years, for whom an increased one-year incidence of any type of cancer was associated with platelet counts in the range of 326–400 × 10^9^/L. In the case of these older males, very high platelet counts of >400 × 10^9^/L were associated with an increased risk of advanced cancer at the time of diagnosis, most commonly lung and colon cancers [1]. The authors contend that their findings “support the usefulness of platelet counts as a clue to identifying patients who could be harbouring a cancer” [1].

In the second of these, a very recently reported Canadian study by Giannakeas and colleagues described the findings of a nested case-control study, which encompassed 8,917,187 participants with a median age of 46.4 years (IQR, 32.5–59.5 years) who were resident in Ontario, the most populated province in Canada [2]. The primary objective of this study was to evaluate the possible association of an elevated platelet count with a cancer diagnosis [2]. Complete blood count data spanning the ten-year period from January 2007 to December 2017, with an additional one-year follow-up, was derived from the ICES, formerly known as the Institute for Clinical Evaluative Sciences, an organization that utilizes state-of-the-art analytical methods and systems to monitor and streamline healthcare delivery and outcomes throughout the province and beyond. The Ontario Cancer Registry provided data with respect to the prevalence and types of solid neoplasms [2]. Participants had one or more complete blood counts performed during the ten-year period of the study. Each case participant who presented with a new cancer diagnosis was also grouped with three matched, cancer-free case-control subjects [2].

Based on the platelet count distribution of the cancer-free controls, platelet counts were grouped into five categories: (i) very low (≤10th percentile); (ii) low (>10th–25th percentile); (iii) medium (>25th–75th percentile); (iv) high (>75th–90th percentile); and (v) very high (≥90th percentile). Of the total cohort, 495,341 (5.6%) participants who had had a complete blood count recorded were diagnosed with their first primary cancer during the ten-year observation period. Relative to those with a medium platelet count measured in the preceding six months, the odds ratio (OR) for the development of a solid malignancy in those participants with a very high platelet count was 2.32 (95% CI, 2.28–2.35) [2]. These very high platelet counts were associated with “colon (OR, 4.38; 95% CI, 4.22–4.54), lung (OR, 4.37; 95% CI, 4.22–4.53), ovarian (OR, 4.62; 95% CI, 4.19–5.09) and stomach (OR, 4.27; 95% CI) cancers” [2]. The authors concluded “that an elevated platelet count could potentially serve as a marker for the presence of some cancer types” [2].

In a parallel-level, retrospective study, the same Canadian research team investigated the possible relationship between platelet count and cancer survival [3]. The study design was based on the identification and recruitment of a sub-group of 112,231 members of the original cohort who had undergone a complete blood count in the 30-day period prior to diagnosis of their primary malignancy. Participants were assigned to one of nineteen different cancer sites, most commonly in the lung, colon, breast and prostate, while liver and hematological malignancies were excluded due to their influence on thrombogenesis [3]. Platelet counts were allocated to one of three categories: low (≤25th percentile), medium (25th–75th percentile) and high (>75th percentile). The median age at cancer diagnosis was 68 years (IQR, 57.8–77.5 years) and patients were monitored for an average of 2.6 years [3].

Of the total cohort, 40,329 patients died from their index cancer during the follow-up period. The rate of cancer-specific deaths was higher among participants with a high platelet count (hazard ratio (HR) 4.52; 95% CI, 1.48–1.55) and was associated with poor survival for many types of cancer, most prominently, and perhaps not surprisingly, colon, lung, ovarian and stomach cancers [3]. Notable exceptions were breast and prostate cancers, for which low platelet counts were associated with worse cancer-specific survival [3]. The authors concluded, “a higher platelet count is associated with cancer-specific death for many common cancer sites … and could potentially be used as a risk stratification measure for intensified treatment” [3].

While the aforementioned works and other earlier studies [4,5] have clearly identified an association of elevated platelet counts with the development and progression of, as well as mortality from, various types of malignancy, lung cancer is particularly prominent in this context. The association between platelets and lung cancer represents the major focus of the remainder of this review, with an emphasis on the following: (i) the association of elevated platelet counts with lung cancer; (ii) the lung as a major site of thrombogenesis; (iii) the major platelet-derived granule protein, TGF-β1, as a key mediator of tumorigenesis, immunosuppression and metastasis, as well as a brief consideration of the pro-tumorigenic activity of another platelet α-granule-derived protein, platelet factor 4, (PF4, CXCL4); (iv) the pro-tumoral activity of platelet-derived MPs; (v) platelets and cancer-associated thrombosis; (vi) the anti-cancer potential of antiplatelet therapies.

## 3. Elevated Platelet Counts and Lung Cancer

In an earlier study, Costantini measured the complete platelet counts of a cohort of patients (*n* = 714) who presented with advanced non-small-cell lung cancer (NSCLC), small-cell lung cancer (SCLC), or colon cancer [6]. The authors noted that, unlike the other types of malignancy, patients with NSCLC manifested an increased prevalence of very high platelet counts (>400 × 10^9^/L), which was maintained during disease progression. The authors attributed their findings to an unusually high level of cytokine-driven bone marrow stimulation in NSCLC, as opposed to a role in disease pathogenesis and progression [6].

However, two subsequent studies revealed that the platelet count is also a determinant of prognosis in NSCLC. In the first of these studies, Yu et al. reported a significant association between the pre-operative platelet count and disease prognosis [7]. This study, which was undertaken in China from 2006 to 2009, involved a cohort (*n* = 510) of recently diagnosed, untreated patients (median age 60 years, range 37–82 years) with operable NSCLC. Patients with more advanced malignancies (T3 and T4, according to the TNM cancer staging system) had significantly higher pre-operative platelet counts than those of patients with stages T1 and T2 [7]. During postoperative follow-up, the respective 3-year cumulative overall survival (OS) probabilities for patients with normal and high pre-operative platelet counts were 75.3% and 59.2%, respectively, while patients in the latter subgroup also had an increased risk of disease progression (HR, 1.568; 95% CI, 1.015–2.2453). The authors concluded that “preoperative platelet counts represent a novel, independent prognostic biomarker in operable NSCLC” [7].

In a somewhat similar study, Ji and colleagues retrospectively investigated possible associations of the pre-operative platelet count with prognosis in a cohort (*n* = 234) of mostly elderly Chinese patients with early stage 1 NSCLC, also undertaken during the period October 2006–December 2009 [8]. The authors reported that patients with an elevated platelet count (*n* = 20 patients, with platelet counts ≥ 300 × 10^9^/L) had a significantly increased risk of disease progression and death, with respective HRs of 5.314 (95% CI, 2.750–10.269, *p* < 0.05) and 3.139 (95% CI, 1.22–8.034, *p* < 0.05).

A later study reported by Zhu et al. is particularly noteworthy [9]. Based on an analysis of the data derived from an earlier high-powered meta-analysis focused on the characterization of putative genomic loci involved in the regulation of megakaryopoiesis and platelet production [10], these authors identified six relatively independent single nucleotide polymorphisms (SNPs) linked to platelet counts [9]. As a strategy to probe the associations between platelet counts and the risk of lung cancer, these six SNPs were selected as instrumental variables (IVs) for inclusion in a Mendelian randomization analysis. Also included in the analysis was Oncoarray information and genome-wide association study (GWAS) data derived from the “International Lung Cancer Consortium and Transdisciplinary Research in Cancer of the Lung” [11]. Multiple IV analyses revealed significant associations between the platelet count and the overall risk of NSCLC. In this context, each increment of 100 × 10^9^/L in the platelet count was associated with a 62% increase in NSCLC risk (*p* = 0.005). In addition, NSCLC subtype analysis revealed a significant association of the platelet count with small-cell lung cancer (*p* = 0.01), while no increase was observed with lung adenocarcinoma or squamous cell cancer, possibly due to insufficient sample sizes. The authors concluded that their findings strengthen the risk between elevated platelet counts and lung cancer, as well as the potential benefit of anti-platelet therapies in the prevention of this malignancy [9].

### Associations between Elevated Circulating Platelet Counts and Subtypes of Lung Cancer

In this context, two recent, extensive systematic review and meta-analysis studies are of note. In the most recent of these, Barlow et al. examined the abstracts from 4766 studies [12]. Twenty of these, encompassing 2974 patients, were deemed eligible, having satisfied the inclusion criteria for meta-analysis, these being an age of ≥18 years, diagnosis with a specific pathological or genetic type of lung cancer and a threshold platelet count of 400 × 10^9^/L [12]. Based on their analyses, the authors concluded that a high platelet count is likely to be an adverse, generic predictor of disease prevalence “across all lung cancer subtypes” [12].

In a prior systematic review and meta-analysis conducted one year earlier, Yuan et al. analyzed 40 studies that encompassed 16,696 lung cancer patients [13]. In this study, disease progression according to OS, disease-free survival (DFS), progression-free survival (PFS) and time to progression (TTP) was monitored in participants with a histologically confirmed diagnosis of lung cancer and a pre-treatment measurement of circulating platelet counts. The authors’ findings revealed that an elevated pre-treatment platelet count was an independent, generic predictor of OS, DFS, PFS and TTP in lung cancer patients [13]. The aforementioned studies, which describe the association of elevated circulating platelet counts with the development and progression of lung cancer, are summarized in Table 1.

In addition to the association with circulating platelet counts per se, another study identified an elevated pre-treatment platelet-to-lymphocyte ratio, as well as low hemoglobin and albumin levels and an elevated C-reactive protein (CRP) level, as being significant independent risk factors for a shorter OS in patients with stage IV NSCLC with malignant pleural effusion [14]. This finding also extended to a subgroup of patients with epidermal growth factor receptor and anaplastic kinase wild-type NSCLC [14].

Although the evidence linking elevated circulating platelet counts to the development of, and decreased survival from, lung cancer is consistent, several studies have failed to detect this association. For example, in an earlier retrospective study encompassing a 3-year period, the prognostic utility of the circulating platelet count measured at the time of diagnosis was investigated in 419 lung cancer patients, of whom 75.3% had stage IIIB/IV disease [15]. The authors of this study did record a 37% decrease in survival of those patients with the highest circulating platelet counts; however, when the tumor stage was included in the covariates, platelet counts were no longer an independent survival factor [15]. In a more recent retrospective study focused on advanced NSCLC to which 135 patients were recruited, 91.1% of whom had stage IV disease, the authors identified decreased circulating lymphocyte counts and high monocyte counts and ferritin levels, but not platelet counts, as being host-related, independent prognostic factors that significantly affected survival rates in patients with NSCLC [16]. Although these two studies are noteworthy, the retrospective nature of both studies, as well as the recruitment of a high percentage of patients with late-stage disease, as opposed to a spectrum of disease stages, may have negated possible associations with the circulating platelet count.

Given the range of factors that may influence circulating platelet counts, including age, gender, ethnicity, genetic variation, co-existent inflammatory disease of infective and non-infective origin, nutritional status, heavy alcohol consumption and others [17], these issues must be taken into consideration when evaluating the reliability of the measurement of circulating platelet counts at a single time point.

## 4. The Lung as a Major Site of Thrombogenesis

Until recently, circulating platelets were believed to originate exclusively from the stem cell/thrombopoietin/megakaryocyte axis in the bone marrow. This concept has, however, been revisited following the seminal studies of Lefrançais and colleagues [18,19]. Using sophisticated intravital microscopy imaging of the pulmonary microcirculation of mice, these investigators identified the lung as a primary site of thrombopoiesis. Their studies revealed the transit of large numbers of bone marrow-derived megakaryocytes through the lungs, where they are trapped, often at vascular bifurcations, releasing large numbers of platelets into the pulmonary microcirculation [18,19]. The authors state that platelet biogenesis in the murine lung accounts for “approximately 50% of total platelet production or 10 million platelets per hour” [18], while estimates of platelet production of 7–100% in the lungs of humans and mice have been proposed by others [19]. Albeit unproven, it is conceivable that the dysregulation of platelet production in the lungs, possibly exacerbated by smoking, may be a significant contributor to lung tumorigenesis.

## 5. The Platelet α-Granule-Derived Immunosuppressive Factor, Transforming Growth Factor-β1

The pro-tumorigenic cytokine, TGF-β1, and the chemokine, PF4 (CXCL4), are not only abundant in the α-granules of platelets but are also expressed by many types of human tumor, including lung cancer [20,21,22]. TGF-β1, in particular, is seemingly a key player in disease pathogenesis.

### 5.1. Transforming Growth Factor-β1

Most mammalian cell types contain one or more of the three isoforms of TGF-β. In humans, platelet α-granules represent the largest reservoir of TGF-β, predominantly of TGF-β1. In addition to platelets, only megakaryocytes, regulatory T cells (Tregs), B-lymphocytes and some types of tumors, including lung adenocarcinomas, possess the machinery to convert stored, latent, inactive TGF-β1 to its biologically active, pro-tumorigenic, immunosuppressive form [23,24,25]. This conversion involves the activation-mediated mobilization of platelet α-granules and the binding, via disulfide and covalent bonds, of the latency-associated peptide of inactive TGF-β1 to the trans-membrane protein, glycoprotein-A repetition predominant (GARP), which is the receptor for latent, secreted TGF-β1 [23]. The dissociation of active cytokine from this complex appears to require the involvement of unknown factor(s) that are present in platelet releasate. However, in the case of Tregs, the binding of the GARP/TGF-β1 complex with membrane αVβ8 integrins results in the presentation and release of active TGF-β1 [24,25]. Tumor cells, on the other hand, do not produce GARP, which is acquired from platelets, Tregs and B-lymphocytes in the tumor microenvironment (TME) [25].

As a result of the predominance of activated platelets and Tregs in the lung cancer TME, as well as the hijacking of GARP by tumor cells, a menacing, TGF-β1-enriched milieu is established, which is likely to contribute to tumor progression, resistance to tumor-targeted therapies and a poor prognosis [26,27,28].

#### 5.1.1. Immunosuppressive Activities of TGF-β1

Transforming growth factor-β1 is a broadly non-specific, immunosuppressive cytokine that targets the cells of both the adaptive and innate immune systems and even co-opts structural cells in the TME, such as cancer-associated fibroblasts (CAFs).

#### 5.1.2. TGF-β1-Mediated Suppression of Adaptive Anti-Tumor Cellular Immune Mechanisms

The inhibitory effects of TGF-β1 on the proliferation and differentiation of CD4^+^ T-lymphocytes of the T helper 1 (Th1) phenotype were observed in several earlier studies. One such study described the suppressive effects of TGF-β1 on the production of the anti-tumor cytokines, interleukin (IL)-2 and interferon (IFN)-γ, via mechanisms that were attributed to a decreased expression of the lineage-determining, pro-Th1 differentiation factor, T-bet (T-box transcription factor 21, TBX21) [29].

In addition to the suppressive effects on Th1 cells, the exposure of murine CD8^+^ cytotoxic T-lymphocytes to TGF-β1 in vitro was found to result in the attenuation of anti-tumor activity, which was associated with interference with the intracellular signaling mechanisms involving the transcription factors, ATF1 (cyclic AMP-dependent transcription factor 1) and Smads (suppression of mothers against decapetaplegic) [29]. This, in turn, resulted in an attenuated expression of the cytotoxic T-lymphocyte-derived mediators of tumor cell apoptosis and anti-proliferative activity, namely, perforin, granzymes A and B, the Fas ligand and IFN-γ [30]. More recent studies have identified additional mechanisms of TGF-β1-mediated interference with the anti-tumor activity of cytotoxic T-lymphocytes. These include the downregulation of expression of the homodimeric C-type lectin receptor, NKGZD, which is present on cytotoxic T-lymphocytes and natural killer (NK) cells, thereby preventing the interaction of this adhesin with its ligand, NKGZDL, as expressed on tumor cells [31]. The intracellular signaling mechanisms underpinning the regulation of the anti-tumor activities of cytotoxic T-lymphocytes appear to involve the induction of Smad 4 via the triggering of MEK/ERK (mitogen-activated protein kinase kinase/extracellular signal-regulated kinase) signaling; however, they are complex and remain incompletely understood [32].

Even more recently, Rafia et al. reported that the exposure of human Vγ9Vδ2 T-lymphocytes to TGF-β1 interfered with the antigen-mediated activation and anti-tumor activity of these cells in vitro [33]. Exposure of these γ/δ T-lymphocytes to the cytokine resulted in phenotypic, transcriptomic and metabolic changes, specifically, the decreased expression of surface receptors that promote interactions with tumor cells, as well as interference with mitochondrial respiration and glycolysis, which resulted in cell death [33].

#### 5.1.3. Immunosuppressive Interactions of TGF-β1 with Regulatory T Cells (Tregs)

Arguably the most prominent, pro-tumorigenic role played by TGF-β1 relates to its critical involvement in promoting the maturation/differentiation, stability, viability and immunosuppressive functions of Tregs [34,35,36]. In the case of NSCLC, the numbers of these cells are not only higher in the TME relative to the adjacent lung tissue but also manifest a significantly increased immunosuppressive phenotype [37]. This distinctive intra-tumoral phenotype is characterized by the upregulated expression of Foxp3 (forkhead box P3) mRNA and protein, as well as significantly increased levels of mRNA encoding four transcription factors, namely, Eos (zinc finger protein Eos), IRF4 (interferon regulatory factor 4), SATB1 (special AT-rich sequence-binding protein) and GATA (GATA-binding factor 1). This phenomenon of “Treg-locking” appears to confer both stability and immunosuppressive function [37].

Prominent immunosuppressive functions of Tregs include: (i) the secretion of the immunosuppressive cytokines, IL-10 and IL-35, as well as TGF-β1 per se [38]; (ii) the constitutive expression of several co-inhibitory immune checkpoints, including CTLA-4 (cytotoxic T-lymphocyte-associated protein 4, CD152), LAG-3 (lymphocyte activation gene-3, CD223), PD-1 (programmed cell death protein 1, CD279) and TIM-3 (T-cell immunoglobulin and mucin domain 3, CD366) [39]; (iii) the expression of two enzymes, ectonucleoside triphosphate diphosphohydrolase (CD39) and ecto-5′-nucleotidase (CD73), which, in turn catalyze the sequential hydrolysis of adenosine triphosphate to adenosine, which, via agonistic interactions with the type-2 adenosine receptors that are widely expressed on cells of the adaptive and innate immune systems, possesses broad-spectrum immunosuppressive activities [40,41]; (iv) removal by antigen-specific Tregs of the corresponding antigenic peptide/MHC class-II complexes presented by pulsed dendritic cells [42].

### 5.2. TGF-β1-Mediated Suppression of Innate Anti-Tumor Cellular Immune Mechanisms

Although the pro-tumorigenic and immunosuppressive properties of TGF-β1 are often mediated indirectly via interactions with Tregs, this cytokine also directly suppresses the anti-tumor activities of cells in the innate system, specifically, the dendritic cells (DCs), NK cells, monocytes/macrophages and neutrophils.

#### 5.2.1. Dendritic Cells

The protective activities of DCs, which are compromised by exposure to TGF-β1, include the failure of maturation and differentiation of these cells, as well as defective antigen presentation due to the attenuation of expression of histocompatibility MHC class-II molecules, as well as the co-stimulatory molecules, CD80 and CD86, resulting from the TGF-β1-mediated induction of the immunosuppressive molecules, indoleamine 2,3-dioxygenase and arginase 1 [43,44,45]. Additional TGF-β1 DC-targeted immunosuppressive mechanisms include the decreased migration of DCs to draining lymph nodes and an impaired ability to stimulate the production of IFN-γ by tumor antigen-primed T lymphocytes [46].

#### 5.2.2. Natural Killer Cells

The IL-15-mediated expansion of NK cells and their anti-tumor effector functions, mediated via antibody-dependent cellular cytotoxicity and the expression of other receptors involved in tumor cell killing, including NKG2D, NKp30 and DNAM-1, are attenuated by TGF-β1. These immunosuppressive effects of the cytokine result from the impairment of Tbet- and mTOR (mammalian target of rapamycin)-driven intracellular signaling mechanisms, resulting in defective degranulation and the release of the anti-tumor agents, granzyme B, perforin and IFN-γ [43,44,47,48].

#### 5.2.3. Macrophages

Active TGF-β1, which is present in high concentrations in the TME, has been reported to promote the autocrine and paracrine transition of uncommitted macrophages to the IL-10-producing M2 phenotype [49]. Although it is not entirely clear, the mechanism of this transition appears to involve the TGF-β1-mediated over-expression of the SNAIL1 transcription factor (zinc finger protein transcription factor 1), which is associated with the involvement of the Smad 2/3 and phosphatidylinositol 3-kinase/AKT pathways [49].

#### 5.2.4. Neutrophils

Similarly, exposure to TGF-β1 in the TME promotes the transition of neutrophils from the anti-tumor N1 phenotype to the pro-tumorigenic N2 phenotype [50], albeit by mechanisms that remain unclear, but appear to involve increased reactivities of the neutrophil pro-oxidative enzyme, myeloperoxidase [51] and the arginine-limiting enzyme, arginase 1 [52].

Collectively, the aforementioned immunosuppressive mechanisms, which are summarized in Table 2, represent a formidable obstacle to effective anti-tumor immunity by subverting the protective activities of a wide range of cells of the adaptive and innate immune systems.

## 6. Effect of TGF-β1 on Cancer Cells Per Se

The protein known as the X-linked inhibitor of apoptosis (XIAP)-associated factor 1 (XAF1) is a tumor-suppressor gene expressed in many types of tumor cells, including lung adenocarcinoma and colon tumor cells [53,54,55]. In the case of human colon cancer cells, exposure to TGF-β1 in vitro resulted in the protection of these cells from apoptosis by a mechanism involving the activation of ERK 1/2 and the repression of transcription of XAF1 [55]. Although seemingly unexplored, this mechanism may also underpin the anti-apoptotic effects of TGF-β1, derived largely from platelets and Tregs in the TME, on lung cancer cells.

In addition to the anti-apoptotic interactions of TGF-β1 with tumor cells, platelet-derived TGF-β1 has also been reported in an earlier study as driving the transition of colon carcinoma and breast cancer cell lines to an invasive, mesenchymal-like phenotype, via the activation of Smad- and NFκB (nuclear factor κB)-dependent mechanisms [56]. An infusion of these cells into experimental animals resulted in the development of metastatic foci in the lungs [57]. The additional mechanisms of platelet-derived TGF-β1-driven epithelial-mesenchymal transition, which are broadly operative in various types of lung cancer cells, include epigenetic modifications, also involving the TGF and NFκB pathways, which result in DNA demethylation of the SNAIL1 and CD87 (urokinase receptor) genes [57].

Notwithstanding their involvement in epithelial-mesenchymal transition, circulating platelets also contribute to metastatic lung cancer and other types of solid malignancy by inducing vascular adhesion molecule expression and the trans-endothelial migration of tumor cells, which have then transitioned to the aggressive mesenchymal phenotype [58,59]. These interactions involve an array of receptors and counter-receptors on platelets, the vascular endothelium and tumor cells. Prominent examples in the case of platelets include glycoprotein VI, FcγRIIa and the C-type lectin-like-2 receptor (CLEC-2) [60].

## 7. Effects of TGF-β1 on Cancer-Associated Fibroblasts

Using an in vivo experimental animal model of collagen-dependent lung cancer metastasis, Wei et al. reported that attenuation of TGF-β1 signaling resulted in the inhibition of SNAIL1 expression and collagen deposition [60]. The cellular targets of inhibition of TGF-β1 signaling were identified as cancer-associated fibroblasts (CAFs) or tumor cells, according to the presence of active lysyl oxidase-like 2 (LOXL2), implicating both cell types cells as major contributors to TGF-mediated pulmonary fibrosis and metastasis in advanced lung cancer [60].

Notwithstanding the pro-fibrotic, pro-tumorigenic actions of pulmonary fibroblasts, these cells have also been reported to express the co-inhibitory immune checkpoint, PD-L1 (programmed death-ligand 1, CD274), which was identified in biopsy specimens taken from patients with idiopathic pulmonary fibrosis (IPF) [61]. Moreover, the expression of PD-L1 by these cells was associated with an invasive phenotype that was found to drive IPF in a humanized murine model of IPF [61]. In a related study, Kang et al. reported that exposure to TGF-β1 induced pro-fibrotic PD-L1 expression in both isolated human IPF fibroblasts and murine fibroblasts [62]. Importantly, TGF-β1-treatment of human lung fibroblasts was found to stimulate the formation of PD-L1-packaged in extracellular vesicles. The release of PD-L1 from these vesicles inhibited the activation of T-lymphocytes by anti-CD3 monoclonal antibodies (mAbs), while mediating fibroblast migration [62].

## 8. Pro-Tumorigenic Activity of Platelet Factor 4

In the case of PF4, this chemokine, in addition to activities that include promoting platelet aggregation and monocyte migration [63], has been shown to induce bone marrow megakaryopoiesis in a murine model of experimental lung adenocarcinogenesis [20]. In this study, the genetically engineered over-expression of PF4 by experimentally induced murine lung adenocarcinomas resulted in the increased accumulation of platelets in the lungs, augmentation of the production of PF4 by these cells and the more rapid progression of the disease, which is indicative of platelet-mediated augmentation of tumorigenesis [20]. In this context, it is noteworthy that relative to matched healthy control subjects (*n* = 463), increased systemic levels of PF4 have been reported in liver transplant recipients who developed lung cancer (*n* = 29; *p* = 0.02), actual PF4 concentrations were not shown, while platelet counts were numerically higher in the lung cancer group (210 × 10^9^/L vs. 179 × 10^9^/L; *p* = 0.08) [64].

Interestingly, in many types of malignancy, there exists a cancer-specific sub-population of circulating platelets, known as tumor-educated platelets (TEPs) [65,66,67]. These TEPs have distinct tumor-specific RNA profiles, resulting, in part, from the differential splicing of megakaryocyte-derived RNA, as well as distinct protein profiles. Tumor-educated platelets, while retaining their hemostatic properties, also manifest a pro-metastatic phenotype [68,69,70]. Although useful in cancer diagnosis, the exact mechanisms of transition of platelets to the TEP phenotype are unclear but appear to involve the systemic exchange of platelet- and tumor-derived biomolecules [70], possibly implying their involvement with TME-derived PF4. This issue clearly merits intensive investigation [68,69,70].

In summary, the preceding two sections have largely focused on the pro-tumorigenic, immunosuppressive activities of the major platelet α-granule constituents, TGF-β1 and, albeit to a lesser extent, PF4. The focus on TFG-β1 and PF4 in the TME does not imply that these pro-tumorigenic mediators are derived solely from platelets, or that they are the only pro-tumorigenic mediators that are present in abundance in platelet granules [71]. In this latter context, IL-8 and CCL5 (RANTES) are intimately involved in the recruitment of myeloid-derived suppressor cells to the TME [72,73]. Platelet expression of the co-inhibitory immune checkpoint, PD-L1, acquired via transfer from tumor cells, has also been identified as another mechanism of immunosuppression that is operative in NSCLC, as well as being an impediment to co-inhibitory immune-checkpoint-targeted therapy [74].

## 9. Pro-Tumorigenic Activity of Platelet Microparticles

Various cell types, upon activation, malignant transformation, stress, or death, release vesicular structures [75]. These vesicles can be derived from outward blebbing of the plasma membrane (MPs), may occur via the endosomal pathway (exosomes), or arise from the membrane blebs of apoptotic cells [75]. Specifically, MPs are vesicles of 0.1–1 µm in size that carry parent-cell derived surface receptors, proteins, nucleic acids and lipids, as well as high levels of membrane phosphatidylserine, some of which may be delivered to target cells [75]. Depending on their cellular origin, various types of MPs have been described, including those emanating from platelets (PMP), monocytes (MMP), endothelial cells (EMP) and tumor cells (TMP) [75,76]. Platelet-derived MPs (PMPs), in particular, are the most abundant MPs in the circulating peripheral blood and account for over 70–90% of all extracellular vesicles [77]. Aside from carrying many membrane transporters and adhesion receptors, PMPs contain a variety of molecules, including transcription, growth and coagulation factors, as well as enzymes, bioactive lipids, cytokines, chemokines, complement proteins and others [78]. Delivery of these factors enables PMPs to interact with leukocytes, endothelial cells and tumor cells, among others [78].

Increased numbers of circulating MPs have been reported in a wide range of diseases, including various malignancies, in which they have been implicated in tumor invasion, angiogenesis, metastasis and coagulation [75,76,79,80]. With respect to lung cancer, several types of circulating MPs, including platelet-derived, activated MPs, are present at increased levels in patients compared to healthy control subjects [81]. These findings were confirmed in a more recent study, which reported that PMPs were not only significantly elevated in lung cancer patients, but also that their levels were significantly higher in those patients with cancer-associated thrombosis (CAT), in comparison to CAT-negative patients, and were highest in those patients with the shortest survival times [82]. The authors speculated that these increased levels of PMPs (as well as those of other biomarkers) were related to CAT in lung cancer patients [82]. In an even more recent study, Liu et al. reported that the circulating levels of both PMPs and neutrophil microparticles (NMPs) were increased in treated, advanced NSCLC patients who had progressive disease, as opposed to those with objective remission [83]. They suggested that combining laboratory indices such as these may predict disease progression in lung cancer [83].

Platelet microparticles may play a role in tumor growth, metastasis and angiogenesis. In the case of angiogenesis, this process consists of various steps, including endothelial cell (EC) proliferation and the directed migration of ECs, as well as tubulogenesis, all of which are regulated by various growth factors and signaling pathways [84]. With respect to the involvement of PMPs, earlier studies reported that these vesicles induce angiogenesis in vitro by promoting proliferation, survival, migration and the capillary tube formation of human umbilical vein ECs (HUVECs) [85]. This transition involved a pertussis toxin-sensitive G protein, ERK, and the phosphoinositide 3-kinase pathway [85]. Moreover, PMPs were also shown to induce angiogenesis in an in vivo model of ischemia, in which these particles were transplanted subcutaneously [86]. Certain growth factors, comprising vascular endothelial growth factor (VEGF), basic fibroblast growth factor (bFGF) and platelet-derived growth factor (PDGF), all appeared to play a role in the upregulation of blood vessel formation by PMPs [86].

Metastasis is a multi-step process encompassing: (i) the escape of cancer cells from the primary tumor; (ii) migration of the cancer cells into vessels; (iii) cell survival in the circulatory system; (iv) extravasation; and (v) proliferation at a distant site, leading to colonization [87,88]. With respect to their involvement in this process, PMPs were shown to interact with lung cancer cells and to enhance the various stages of metastasis, as well as demonstrating angiogenesis in vitro [89]. In this setting, PMPs interacted with lung cancer cell lines to cause: (i) the migration of these cells; (ii) increased adhesion to ECs and fibrinogen by transference mediated by platelet-derived integrins such as CD41; and (iii) increased proliferation, which was associated with the activation of the MAPK p42/44 and AKT signaling pathways [89]. In addition, the matrix metalloproteinases (MMP-2, MMP-9 and MT1-MMP), which play a role in the catalytic breakdown of extracellular matrix components, were upregulated in some, but not all, of the cancer cell lines investigated, and were associated with increased chemoinvasion across Matrigel matrices [89]. Moreover, the expression of several genes involved in tumor vascularization, such as VEGF, IL-8 and hepatocyte growth factor (HGF)) was also upregulated following exposure to PMPs. Finally, it was shown that the intravenous injection of PMP-coated Lewis lung carcinoma (LLC) cells into mice resulted in significantly more metastatic foci in the lungs, as well as the presence of LLC cells in the bone marrow, relative to the control mice [89].

The contributions of PMPs to the clinical manifestations of cancer are likely to be substantial, but they remain poorly understood because of difficulties in the selective separation of platelet vesicles in the absence of contamination of vesicles derived from other cell types [77]. Clearly, more sophisticated studies based on cancer models in vitro, as well as clinical studies, are necessary to further elucidate the role of PMPs in cancer [77].

## 10. Cancer-Associated Thrombosis

Platelets have been implicated in the pathogenesis of the pro-coagulant state in cancer, a setting in which cancer cells directly activate platelets and enhance thrombus formation [90,91]. Indeed, cancer patients often develop thrombotic complications, such as deep vein thrombosis (DVT) and pulmonary emboli (collectively referred to as venous thromboembolism (VTE)) or arterial thrombosis [91]. This hypercoagulable state, also known as Trousseau’s syndrome, was first described by Dr. Armand Trousseau nearly 150 years ago, when he observed that migratory thrombophlebitis is a diagnostic element of visceral cancer [92]. Importantly, thrombotic events are the second most frequent cause of death in cancer patients, only after disease progression itself [93], with cancer-related VTE having been shown in the Framingham Heart Study (USA) to be associated with a 16-fold higher risk of death than in those patients without VTE [94]. Although it is uncertain as to whether VTE was merely a symptom of advanced cancer in this study, the diagnosis of thrombosis in patients with cancer is clearly a poor prognostic sign [94,95].

Different types of cancer carry a variable risk of VTE [96]. In the context of lung cancer, epidemiological studies suggest that this malignancy belongs to a group of cancers with the highest incidence rates of VTE [97]. Indeed, the risk of thrombosis in lung cancer was found to be 100-fold higher than in the general population, posing a significant health problem in these patients [98], with incidence rates of between 3 and 13.9% of thromboembolic events having been reported in lung cancer study groups during the period of 2008–2014 [99,100,101,102,103].

A more recent cohort study, comprising 10,598 patients with lung cancer, found an overall VTE incidence of 39.2 per 1000 person-years [104]. Independent factors associated with the increased risk of VTE included metastatic disease and the adenocarcinoma subtype. This study also reported an approximately 50% higher risk of mortality in patients with VTE [104]. Another prospective, global, cohort study reported an incidence of VTE of 6.1% among those lung cancer patients who were beginning a new cancer therapy [105]. Of these, 47%, 46%, 4% and 3% developed pulmonary embolism, deep vein thrombosis, visceral thrombosis, and catheter-associated thrombosis, respectively [105]. A much higher incidence rate of DVT (18.75%) was reported among 160 lung cancer patients in a very recent retrospective study by Jin et al. [106]. Adenocarcinoma, was the most frequent (90%) type of cancer, while risk factors for DVT included advanced disease, more severe myocardial injury and a hypercoagulable state [106].

In several other studies, adenocarcinoma and advanced disease were recognized as important risk factors for VTE in lung cancer. Other risk factors included NSCLC (versus SCLC), pneumonectomy and the use of specific chemotherapeutic drugs, including anti-angiogenic agents, as well as elevated pre-chemotherapy platelet counts [97].

Other types of cardiovascular disease (CVD)-related mortality that occur in NSCLC have been described in a retrospective cohort study, as reported by Kravchenko et al. [107]. These authors investigated the association between mortality and seven types of CVD, both individually and in combination, detected at the time of diagnosis of various stages of NSCLC and during the period of treatment and follow-up (1992–2007) in older patients (*n* = 95,167) aged >65 years. The most prominent causes of CVD-related death during follow-up were comorbid heart failure, myocardial infarction and cardiac arrhythmias [107].

Although this section of the review has focused on the risk that is posed predominantly by lung cancer regarding the development of CVDs, it is noteworthy that the opposite is also the case, such that those with pre-existing CVDs are at high risk for the development of various types of malignancy, including lung cancer [108]. In this context, in a retrospective, population-based cohort study that covered the period 2004–2015, Batra et al. investigated the impact of pre-existing CVDs on treatment patterns and survival outcomes in lung cancer patients (*n* = 20,689; median age of 70 years at diagnosis) [109]. One-third of the patients had at least one CVD, most commonly, congestive heart failure, which was present in 15% of patients. The authors reported that these patients were unlikely to receive any type of anti-cancer therapy and had a higher risk of non-cancer-associated mortality [109]. Even more recently, a population-based, matched cohort study was conducted, based on data derived prospectively from the Danish national registries, spanning the period from 1967 to 2006 and encompassing 306,285 patients with, and 1,222,140 matched participants without, various types of CVD. Wang et al. studied the data to investigate the association of pre-existing CVDs with the subsequent development of, and mortality from, lung cancer [110]. The authors concluded that patients with CVDs, most prominently those with cardiac, vascular and hypertensive disease, had a 67% increased risk of development of lung cancer and an associated 95% increased risk of lung cancer mortality [110]. However, none of these important studies addressed the roles of platelet numbers and activation status in the pathogenesis of these adverse events [108,109,110].

Notably, as a strategy to prevent severe toxicity and the worsening of disease in patients with advanced (unresectable stage 3) NSCLC and pre-existing CVDs, the modification of current anti-cancer therapy (concurrent platinum-based chemoradiotherapy (CRT) followed by maintenance immunotherapy with adjuvant durvalumab) has been proposed. This strategy involves the sequential administration of CRT, together with CVD-targeted therapy combining a beta-blocker with a statin [111].

### Possible Mechanisms of Platelet Activation in CAT

Although platelets play key roles in maintaining homeostasis and contributing to anti-infective innate immune mechanisms, the inappropriate activation of these cells in the TME and circulation appears to drive a thrombogenic phenotype. As mentioned above, the main reason for the cancer-associated hypercoagulable state and high thrombotic risk in cancer patients is that tumor cells can interact with and activate platelets, although other cell types and molecules may also be involved [112]. Cancer cells express and secrete platelet agonists in the TME, causing platelet activation locally and systemically, driving tumor cell-induced platelet aggregation (TCIPA) [112,113]. These events are directly related to the pro-thrombotic state in cancer, while also playing a role in tumor growth and metastasis [114]. In this context, the cytokine, IL-6, derived from tumor cells [115,116], not only drives thrombopoiesis [117] but also promotes platelet activation [118]. These activated, pro-aggregatory platelets may, in turn, enter the circulation during tumor cell dissemination, where they may also contribute to thrombogenesis.

Tumor cell-induced platelet aggregation using different cancer cell lines has been demonstrated in several in vitro studies, while a body of evidence also shows that platelet activation is increased in vivo in patients with cancer [113]. Various mechanisms of tumor-induced platelet aggregation have been described, which vary among different types of tumors. These mechanisms include tumor cell-induced thrombin generation, resulting in the release of adenosine diphosphate, thromboxane A2 and MMP-2 from platelets [119]. With respect to TCIPA, platelets aggregate around the cancer cells, protecting the circulating tumor cells from the deleterious effects of shear forces, as well as from the immune system, by forming a physical barrier around these cells [120]. These actions may contribute to metastatic spread and tumor growth [120].

Other mechanisms that can stimulate clotting and thrombosis in cancer include the release of tissue factor (TF) by tumor cells and, as mentioned above, by tumor-derived MPs that stimulate the extrinsic coagulation cascade, resulting in the activation of factor X, leading to thrombin formation, fibrin synthesis and platelet activation [96,121]. Consequently, TF can promote thrombosis and has, indeed, been found to be predictive of VTE in pancreatic cancer [122]. In the context of lung cancer, levels of TF have been found to be upregulated in the lung tissue and plasma of NSCLC patients [123], with an up to 41-fold increase in levels reported in the case of a giant-cell lung carcinoma patient with Trousseau’s syndrome [124]. High levels of TF were also reported in a case study described earlier by Sato et al. (2006) [125]. The authors suggested that TF originating from lung cancer appeared to be responsible for recurrent DVT/PE (pulmonary embolism) in a patient with Trousseau’s syndrome [125]. Other aspects have more recently been addressed, including the finding that in NSCLC, a high expression level of TF is associated with worse overall survival [126]. In addition, a pathogenic role of TF in tumor growth, metastasis and invasion in NSCLC has been described [126]. With respect to the TF pathway inhibitor-1 (TFPI-1), the main inhibitor of the extrinsic coagulation pathway, it has been reported that NSCLC patients with DVT or metastasis had significantly lower levels of this inhibitor than those without DVT or metastasis [127].

Other mediators released by cancer cells that can cause platelet activation include, firstly, cancer pro-coagulant, a protease that activates factor X independently of TF, and secondly, the enzyme heparanase, which is found in platelets and enhances TF activity [95]. The release of the latter enzyme induces a positive feedback cycle of increased TF activation, which then activates the platelets, leading to increased release of heparanase [95]. In this context, heparanase-mediated pro-coagulant activity has been found to be increased in patients with NSCLC [128].

In addition to the above, tumor cells (as well as platelets and ECs) may also release the plasminogen activator inhibitor type I (PAI-1), which inhibits fibrinolysis and promotes coagulation, actions that may increase the risk of thrombosis in cancer [129] as well as stimulating tumor growth, cancer cell survival and metastasis [130].

Tumor-cell-derived podoplanin (PDPN) is also a key regulator of platelet activation and aggregation [91]. Podoplanin is a type I transmembrane sialomucin-like glycoprotein that is located in certain types of human cancers, including squamous cell carcinoma, brain cancer, glioblastoma, osteosarcoma, bladder cancer and others (in studies cited by Miyata et al., 2017) [131], as well as several normal tissues. Podoplanin induces platelet activation by binding to CLEC-2, thereby facilitating cancer metastasis and cancer-associated thrombosis [132]. In this context, CLEC-2 is a type II transmembrane receptor that is highly and selectively expressed in human platelets and megakaryocytes, as well as the Kupffer cells, albeit at lower levels in the case of the latter [132]. The extracellular domain of PDPN contains platelet-aggregation-stimulating (PLAG) domains, which bind CLEC-2 on the surface of platelets [132].

Hwang et al. [133] cited several studies in human and murine models, which highlight: (i) the crucial role of PDPN in TCIPA [134]; (ii) the strong association between PDPN-mediated TCIPA and the incidence of VTE in cancer patients [135]; and (iii) the association of PDPN with the occurrence of TCIPA, tumor growth and metastasis [131,136]. Furthermore, the involvement of CLEC-2 in the hematogenous metastasis of PDPN-producing cancer cells and cancer-associated thrombosis has been demonstrated in a murine model of experimental carcinogenesis [137]. The aforementioned studies, as well as others, clearly underscore the involvement of the CLEC-2-PDPN axis in platelet–tumor cell interactions [133].

The aforementioned mechanisms by which tumor- and platelet-derived factors may promote clotting and thrombosis in lung cancer are summarized in Table 3.

This section of the review has highlighted the critical role of platelets in cancer-associated vascular endothelial dysfunction, which underscores the potential utility of platelet-targeted therapies in cancer, particularly lung cancer.

## 11. Platelet-Targeted Therapies

Notwithstanding the benefits of aspirin in the treatment of hereditary colorectal cancer, as well as early-onset conventional and advanced adenomas [138,139,140,141,142], confirmation of the potential benefit of antiplatelet agents in the therapy of lung cancer and other types of malignancy remains inconclusive [140,141]. In this context, it is concerning that the use of dual antiplatelet therapy (aspirin + a P2Y12 receptor antagonist, such as clopidogrel or ticagrelor), either without or with the addition of adjunctive vorapaxar, an antagonist of the thrombin-activated PAR1 receptor, [143], poses the threats of increased bleeding and toxic interactions with anti-cancer drugs [141,144,145].

There are, however, several alternative platelet-targeted strategies that show promise in the adjunctive treatment of lung cancer.

### 11.1. Strategies Aimed at Decreasing Circulating Platelet Counts

Certain strategies are focused on decreasing platelet numbers in the setting of the retention of normal platelet function. They include the targeting of IL-6, a key driver of the hepatic synthesis of thrombopoietin [117], with humanized mAbs such as tocilizumab. In this context, it is noteworthy that systemic levels of IL-6 are increased in various types of severe lung cancer and are not only predictive of disease progression [146] but also of an unsatisfactory response to PD-1-targeted immunotherapy in NSCLC [147].

Preliminary findings from a preclinical murine model of experimental therapy have also indicated the potential of administration of thrombopoietin-targeted anti-sense oligonucleotides in decreasing platelet, megakaryocyte and megakaryocyte progenitor counts, with the retention of function [148].

### 11.2. Platelet Adhesion Molecules as Potential Targets

Targeting of the platelet adhesion molecules CD62P (P-selectin), the heterodimeric integrin, GPIIb/IIIa, CLEC-2 and GPVI, with either small-molecule antagonists or humanized mAbs, also has therapeutic, albeit unproven, potential [141,149].

### 11.3. Transforming Growth Factor β1

The targeting of TGF-β1, which is abundant in platelet α-granules, represents another strategy that not only regulates the pro-tumorigenic activity of these cells but also attenuates the resistance of lung cancer to PD-1/PD-L1-directed immunotherapy [150,151,152]. Importantly, in this context, advanced lung cancer is associated with elevated systemic levels of TGF-β1 [153,154].

Promising TGF-β1-targeted strategies include, firstly, the administration of fresolimumab, a humanized mAb, which targets all three isoforms of TGF-β1. Fresolimumab is currently undergoing phase II clinical evaluation in combination with stereotactic ablative radiotherapy as a treatment for early-stage (1A–1B) NSCLC (NTC 02581787) [155]. Secondly, the bifunctional fusion protein, bintrafusp alfa, which enables dual targeting of the TGF-βRII receptor and PD-L1. This innovative, anti-cancer immunotherapeutic agent has recently undergone stringent efficacy and safety testing in a global, open-label phase I trial in adults (*n* = 83) with advanced NSCLC who had progressed following chemotherapy [156]. Although the primary endpoint of the best overall response was not achieved, the authors reported that “some clinical activity and a manageable safety profile” was achieved, with four and nine patients manifesting partial responses and stable disease, respectively [156]. Bintrafusp alfa is currently undergoing phase III clinical evaluation as a first-line treatment in patients with advanced NSCLC with a high-level expression (≥50%) of PD-L1, using pembrolizumab as a comparator (NCT03631706) [157]. This study may determine if this agent will be clinically useful in the treatment of lung cancer.

Additional TGF-β-targeted strategies include the administration of small-molecule pharmacological inhibitors of the receptor-linked enzyme, TGF-β receptor kinase. Currently, more than ten of these agents are undergoing pre-clinical or clinical evaluation [151], predominantly as anti-fibrotic agents in non-malignant disorders. However, given the protective, pro-fibrotic conditions that are prevalent in the TME, these agents, in addition to possessing immunostimulatory activities, may also increase the exposure of tumor cells to cytotoxic T-lymphocytes and NK cells by inhibiting the tumor synthesis of membrane-associated, extracellular matrix proteins. These agents may, therefore, have adjunctive anti-cancer potential; however, extensive clinical development is required.

## 12. Conclusions

The association between elevated circulating platelet counts and the development, progression and adverse outcomes of various types of malignancy is well recognized, most prominently in lung cancer, possibly due to the lung being a major site of thrombopoiesis. The link between lung cancer and thrombopoiesis may also underpin the ominous predisposition of those afflicted with this malignancy to the development of thrombotic disease. Despite concerns about the safety of conventional pharmacological inhibitors of platelet activation as adjuncts to anti-cancer therapy, several alternative strategies, which are in the early pre-clinical stages of design and development, show promise. These include strategies aimed at decreasing platelet counts in the setting of the retention of normal homeostatic function. In addition, the targeting of TGF-β1, one of the most prominent platelet-derived, pro-tumorigenic cytokines, with mAbs and/or small molecule antagonists also shows early promise.

## Figures and Tables

**Table 1:** Summary of studies that describe the association of elevated circulating platelet counts with the development and progression of lung cancer.

Type of Study	Purpose	Outcome	Reference
Prospective cohort study	To determine the relationship of the platelet count with the one-year incidence of cancer of any type (*n* = 295,312 participants aged >40 years, with no prior cancer diagnosis).	The one-year incidence of cancer of any type was associated with elevated platelet counts of 326–400 × 10^9^/L and was highest in males of age >60 years. Lung and colon cancer rates were highest in older males with counts of >400 × 10^9^/L.	[1]
Nested case-control study	Evaluation of an elevated platelet count with a cancer diagnosis (*n* = 8,917,187 participants, median age 46.4 years with no prior cancer diagnosis).	Very high platelet counts (≥90th percentile) measured in the preceding 6 months of diagnosis were associated with a significant risk of development of colon, lung, ovarian and stomach cancers.	[2]
Prospective cohort study	Early study evaluating platelet counts in patients (*n* = 714) with advanced NSCLC, small-cell carcinoma of the lung and colon cancer.	Patients with NSCLC presented with elevated platelet counts of >400 × 10^9^/L, which were maintained during disease progression.	[6]
Retrospective clinical analysis	Evaluation of platelet count in relation to disease prognosis in recently diagnosed, untreated patients with operable NSCLC (*n* = 510, median age 60 years).	Patients with more advanced disease (stages T3 and T4) had higher platelet counts (263 × 10^9^/L and 253 × 10^9^/L, respectively) and an increased risk of disease progression.	[7]
Retrospective clinical analysis	Evaluation of the association of the pre-operative platelet count with prognosis in elderly patients with stage 1 NSCLC.	Patients (*n* = 20) with an elevated platelet count of >300 × 10^9^/L had a significantly increased risk of disease progression and death.	[8]
Retrospective Mendelian randomization analysis	Based on the identification of 6 SNPs linked to platelet counts; these SNPs were selected as instrumental variables in a Mendelian randomization analysis to probe the association between platelet counts and the risk of lung cancer of all types.	Each increment of 100 × 10^9^/L in the platelet count was associated with a 62% increase in NSCLC risk. A significant association with small-cell lung cancer was also detected, but not with lung adenocarcinoma or squamous cell carcinoma.	[9]
Systematic review and meta-analysis	Encompassing 20 studies (*n* = 2974 patients) with lung cancer of all types and a threshold platelet count of 400 × 10^9^/L.	A high platelet count is seemingly a negative, generic predictor of disease prevalence across all cancer subtypes.	[12]
Systematic review and meta-analysis	Analysis of 40 studies that encompassed 16,696 lung cancer patients to evaluate the association of pre-treatment platelet count with disease survival.	An elevated pre-treatment platelet count was found to be an independent, generic predictor of OS, DFS, PFS and TTP in lung cancer patients.	[13]

NSCLC (non-small-cell lung cancer); OS (overall survival); DFS (disease-free survival); PFS (progression-free survival); TTP (time to progression); SNP (single nucleotide polymorphism).

**Table 2:** Pro-tumorigenic, immunosuppressive activities of transforming growth factor-β1.

Adaptive Immunity		
Cell Type	Mechanism	References
CD4^+^ Th1 cells	Decreased production of anti-tumor IL-2 and IFN-γ, which is associated with the decreased expression of T-bet.	[29]
CD8+ cytotoxic T cells	Achieved by several mechanisms: –decreased expression of perforin, granzymes A and B, the Fas ligand and IFN-γ via inhibition of the transcription factors, ATF1 and Smads; –downregulation of the receptor NKGZDL, seemingly via the triggering of MEK/ERK and the induction of Smad4.	[29,30,31,32]
Vγ9Vδ2 T cells	Achieved by a decreased expression of the receptors that interact with tumor cells, as well as by interference with respiration and glycolysis, which precede cell death.	[33]
Regulatory T cells (Tregs)	A key pro-tumorigenic role played by TGF-β1, which involves promoting the maturation, differentiation, stability, viability and immunosuppressive functions of Tregs.	[34,35,36]
Innate Immunity		
Dendritic cells	Failure of maturation of these cells, causing impaired antigen presentation, which is mediated via the production of indoleamine-2,3-dioxygenase and arginase 1.	[43,44]
Natural killer cells	Attenuation of the IL-15-mediated expansion of NK cells, as well as expression of the receptors involved in tumor cell killing, NKG-2D, NKp30 and DNAM-1. These effects result from the TGF-β1-mediated inhibition of Tbet- and mTOR-driven signaling mechanisms.	[43,44,47,48]
Macrophages	TGF-β1 promotes the transition of uncommitted macrophages to the IL-10-producing M2 phenotype by a mechanism involving over-expression of the SNAIL1 transcription factor.	[49]
Neutrophils	TGF-β1 promotes the transition of neutrophils of the anti-tumor N1 phenotype to the pro-tumorigenic N2 phenotype, characterized by increased immunosuppressive activities involving myeloperoxidase and arginase 1.	[50,51,52]

T-bet (T-box transcription factor TBX21); ATF-1 (cyclic AMP-dependent transcription factor 1); Smads (suppression of mothers against decapetaplegic); MEK/ERK (mitogen-activated protein kinase kinase/extracellular signal-regulated kinase); mTOR (mammalian target of rapamycin); SNAIL1 (zinc finger protein transcription factor).

**Table 3:** Tumor- and platelet-derived factors that may stimulate clotting and thrombosis in lung cancer.

Factors	Action	In Vivo Effects in Cancer	References
Tumor-derived tissue factor (TF)	Stimulates the extrinsic coagulation cascade, resulting in the activation of factor X, leading to thrombin formation, fibrin synthesis and platelet activation.	TF was upregulated in the lung tissue and plasma of NSCLC patients; TF originating from lung cancer appeared responsible for recurrent DVT/PE; a lung cancer (LC) patient that presented with several thrombotic events had a 41-fold increase in TF; LC patients with a high TF expression had worse overall survival. Decreased level of the TF pathway inhibitor-1 (TFPI-1) was associated with DVT and tumor metastasis in NSCLC patients.	[96,121,123,124,125,126,127]
Tumor-derived cancer pro-coagulant	Protease that activates factor X, independent of TF.		[95]
Platelet-derived heparanase	Enhanced TF activity	Heparanase-mediated pro-coagulant activity increased in patients with NSCLC.	[95,128]
Tumor- and platelet-derived PAI-1	Inhibits fibrinolysis and promotes coagulation	Pro-tumorigenic function in cancer progression and metastasis	[130]
Tumor cell-derived PDPN	Key regulator of platelet activation and aggregation. Induces platelet activation by binding to CLEC-2 on platelets.	A strong association between PDPN-mediated TCIPA and incidence of VTE in brain cancer and the occurrence of tumor growth and metastasis.	[91,131,135,136]

CLEC-2 (C-type lectin-like receptor 2); DVT (deep vein thrombosis); LC (lung cancer); NSCLC (non-small-cell lung cancer); PAI-1 (plasminogen activator inhibitor type 1); PDPN (podoplanin); PE (pulmonary embolism); TCIPA (tumor-cell-induced platelet aggregation); TF (tissue factor); TFPI-1 (TF pathway inhibitor-1); VTE (venous thromboembolism).

## Data Availability

Not applicable.
